# Maladaptive personality traits may link childhood trauma history to current internalizing symptoms

**DOI:** 10.1111/sjop.12830

**Published:** 2022-05-23

**Authors:** Bo Bach, Sune Bo, Erik Simonsen

**Affiliations:** ^1^ Psychiatric Research Unit Center for Personality Disorder Research, Region Zealand Slagelse Denmark; ^2^ Department of Child and Adolescent Psychiatry Region Zealand Roskilde Denmark; ^3^ Faculty of Health and Medical Sciences University of Copenhagen Copenhagen Denmark

**Keywords:** Childhood maltreatment, personality traits, ICD‐11, emotional disorder, DSM‐5 alternative model of personality disorders (AMPD)

## Abstract

Research supports a strong relationship between childhood maltreatment and internalizing psychopathology (e.g., anxiety and depression), and features of personality are assumed to explain some of this relationship. In this study, we proposed a model in which maladaptive traits mediate the effect of childhood trauma history on internalizing symptoms in adult individuals. A mixed sample (*N* = 462) composed of 142 psychiatric patients and 320 community‐dwelling individuals completed the Childhood Trauma Questionnaire (CTQ), the Personality Inventory for DSM‐5 (PID‐5), and the Symptom Checklist (SCL‐27) for internalizing psychopathology. The effect of childhood traumas explained 34% of the variance in internalizing symptoms while controlling for the influence of age and gender. The traits accounted for 78% of this effect, which was predominantly exerted through the domains of Negative Affectivity, Detachment, and Psychoticism, and specifically through the facets of Depressivity, Suspiciousness, Anxiousness, Perceptual Dysregulation, and Distractibility. This finding provides preliminary support for the proposed model indicating that the aforementioned maladaptive trait domains potentially function as mediating links by which childhood traumas are translated into internalizing symptoms in adulthood. However, these findings must be interpreted with caution due to the cross‐sectional and retrospective mono‐method design of this study. Clinical implications are discussed in relation to transdiagnostic treatment and the potential value of specifying trait domain specifiers in ICD‐11 and DSM‐5 models of personality disorders.

## INTRODUCTION

In the Risk and Prognostic Factors sections of the *Diagnostic and statistical manual* (DSM‐5), childhood trauma is highlighted as a putative etiological contributor to a range of mental disorders, in particular internalized mental health symptoms characterized by internal suffering such as anxiety and depression (APA, 2013). Likewise, ICD‐11 highlights childhood adversity as a risk factor for development of personality disturbances and other mental disorders (WHO, 2022). Accordingly, there is compelling meta‐analytic evidence for a relationship between childhood traumas and internalizing disorders in adulthood (Kendler & Gardner, 2011; McKay, Cannon, Chambers *et al*., 2021), whereas possible causal mechanisms are still not adequately understood. A vast amount of research supports that childhood traumas contribute to the development of trait‐like vulnerabilities (Steele, Townsend & Grenyer, 2019; Torgersen, 2009), which in turn have been found to predict the onset, course, and recurrence of internalizing mental disorders (Alnaes & Torgersen, 1997; Fricke, Moritz, Andresen *et al*., 2006; Gunderson, Stout, McGlashan *et al*., 2011). Recent studies particularly support the association between childhood trauma and maladaptive traits (Bach & Fjeldsted, 2017; Boland, Rock, Johnson, Jones, Salekin & Anderson, 2021; Borroni, Somma, Krueger *et al*., 2019; Granieri, Guglielmucci, Costanzo, Caretti & Schimmenti, 2018; Hemmati, Newton‐Howes, Falahi, Mostafavi, Colarusso & Komasi, 2021). This suggests that childhood traumas may affect the risk for internalizing psychopathology by being a determinant of trait‐like liabilities, such as maladaptive personality traits.

### The role of personality traits

Behavioral genetics attribute 30%–50% of the variance in personality trait expression to genetic factors (Plomin, DeFries, Knopik & Neiderhiser, 2016). Research has attempted to account for the remaining 50%–70% of variance through a range of putative biological and developmental contributors including parental attachment, emotional regulation, and self‐image (Cicchetti, 2016). It has traditionally been theorized that childhood adversities are translated into adult psychopathology by means of maladaptive personality traits that involve vulnerability to psychopathology. For example, Richard von Krafft‐Ebing (1840–1902) viewed the influences of hereditary and environmental factors on mental disorders as often mediated through what he called a ‘neuropathic personality’ (Engstrom & Kendler, 2013). Millon, Grossman, Millon, Meagher and Ramnath (2004) stated that a disordered personality is developed throughout childhood causing an enduring potential for psychopathology in terms of a vulnerable ‘psychological immune system.’ In support of such ideas, research has demonstrated that Personality Disorder (PD) criteria mediate the cross‐sectional relationship between childhood adversity and internalizing disorders (Johnson, Quigley & Sherman, 1997; Kounou, Bui, Dassa *et al*., 2013). Likewise, the mediating role of personality traits has been supported in both cross‐sectional (Enns, Cox & Larsen, 2000) and longitudinal studies (Rodgers, 1996; Schulz, Beblo, Ribbert *et al*., 2017; Spinhoven, Elzinga, Van Hemert, de Rooij & Penninx, 2016), and even when the supposed genetic effect on both personality and psychopathology is accounted for (Keyes, Eaton, Krueger *et al*., 2012; Roy, 2002). For example, a prospective twin‐study by Kendler and Gardner (2011) showed that personality traits mediate the link between risk factors (i.e., genes and childhood environmental stressors) and symptoms of depression and anxiety. Across the identified studies, features of Neuroticism (Gallardo‐Pujol & Pereda, 2013) and low Extraversion (Shu, Chang, Lee *et al*., 2007) accounted for a substantial amount of this mediating effect. Finally, using a non‐clinical sample, Veith, Russell and King (2017) found DSM‐5 maladaptive traits of Negative Affectivity and Detachment to mediate the effect of childhood maltreatment on internalized symptoms.

### The current study

The goal of this study was to test a preliminary mediational model in which trait domains, as measured with the Personality Inventory for DSM‐5 (PID‐5), mediate the relationship between childhood trauma history and adult internalizing symptoms. This was approached by analyzing the cross‐sectional meditational effects of five PID‐5 trait domains (i.e., Negative Affectivity, Detachment, Antagonism, Disinhibition, and Psychoticism), simultaneously, while also comparing the effects of these trait domains against one another.

Because most previous research has highlighted features of Neuroticism and low Extraversion as primary mediators of the relationship in question (Huppert, Abbott, Ploubidis, Richards & Kuh, 2010; Veith *et al*., 2017), we specifically expected the corresponding DSM‐5 trait domains of Negative Affectivity and Detachment to involve significant mediating effects. Additionally, we expected the domain of Psychoticism to comprise a significant mediator due to its substantial relationship with childhood trauma (Back, Flechsenhar, Bertsch & Zettl, 2021). To the best of our knowledge, such mediational effects of DSM‐5 maladaptive traits have not yet been investigated using a clinical sample. Moreover, four of the five DSM‐5 trait domains (i.e., Negative Affectivity, Detachment, Antagonism, and Disinhibition) also serve as trait domain specifiers in the ICD‐11 PD classification, which further underscores the potential relevance of the present study for WHO member states using the ICD‐11 system.

## METHOD

### Participants and procedure

A mixed sample of Danish adults (*N* = 462) comprising 142 clinical and 320 community‐dwelling participants was included (see sample characteristics in Table 1). The clinical participants were recruited from a Danish psychiatric outpatient unit. As a routine segment of their clinical assessment program all patients were consecutively included in the study, and consented to participate in the study. All clinical participants met the criteria for at least one DSM‐IV mental disorder, based on the Mini International Neuropsychiatric Interview (MINI), which is a structured diagnostic interview (Sheehan, Lecrubier, Sheehan *et al*., 1998). Internalizing disorders were most common in terms of anxiety disorders as well as lifetime or current major depressive disorder. Clinical participants suspected of having a substance induced condition, an organic disorder, a current psychotic disorder, a severe depression or autism were not included but referred to another specialized clinical setting.

**Table 1:** Sample characteristics

	Total (*N* = 462)	Clinical (*n* = 142)	Community (*n* = 320)
Gender; *n* (*%*)			
Female	359 (77.7%)	97 (68.3%)	262 (81.9%)
Male	103 (22.3%)	45 (31.7%)	58 (18.1%)
Age; years			
Mean (*SD*)	29.3 (8.4)	29.2 (8.4)	29.5 (8.5)
Range	18–56	18–56	18–56
Relationship status; *n* (%)			
In a relationship	301 (65.2%)	78 (54.2%)	223 (69.7%)
Single	161 (34.8%)	64 (45.8%)	97 (30.3%)
Occupational status;^a^ *n* (%)			
Employed	311 (67.0%)	24 (16.9%)	287 (89.7%)
Unemployed	151 (33.0%)	118 (83.1%)	33 (19.3%)
Educational level; *n* (%)			
Above bachelor level	87 (19.1%)	7 (4.2%)	80 (25.3%)
At bachelor level	106 (22.9%)	9 (6.3%)	97 (30.3%)
Below bachelor level	127 (27.9%)	55 (38.8%)	72 (22.5%)
No education	142 (30.1%)	72 (50.7%)	70 (21.9%)
Lifetime suicidality; *n* (%)			
Never had any suicidal ideations	226 (48.9%)	17 (12%)	207 (64.7%)
Have contemplated suicide	236 (51.1%)	42 (29.6%)	92 (28.7%)
Have planned suicide	102 (22.0%)	13 (9.2%)	2 (0.6%)
Have attempted suicide	88 (19.0%)	70 (49.3%)	19 (5.9%)
Mental health care utilization;^b^ *n* (%)			
Lifetime	298 (64.5%)	142 (100%)	156 (48.8%)
Never had	164 (35.5%)	0 (0%)	164 (51.2%)

^a^ Student, employee, or self‐employed.

^b^ School psychologist, treatment for substance/alcohol abuse, private psychotherapy, psychiatric treatment, or mental health care in general medical practice.

The community‐dwelling participants were recruited using the Danish Civil Registration System. First, we extracted a sample of 1,250 randomly selected local community‐dwelling citizens, which were sent a personal invitation letter (22 addresses proved out‐dated). A total of 351 citizens agreed to participate and were subsequently e‐mailed a secure link to the online assessment procedure, which eventually was completed by 320 participants.

All data were collected via a secure online questionnaire platform (SurveyXact), which prevented missing data. All 462 participants provided informed consent. As a part of their treatment program all clinical participants were given individual feedback on their personality trait profiles. As incentive for participation, the community‐dwelling participants were offered feedback on their personality trait profiles per e‐mail. The study protocol was notified to the Danish Data Protection Agency (SJ‐PSY‐01) and pre‐approved by the Regional Ethics Committee of Zealand. Some of the data and procedures used in the present study were also employed in other already published studies (Bach, Anderson & Simonsen, 2017; Bach & Fjeldsted, 2017; Kongerslev, Bach, Rossi *et al*., 2019). Nevertheless, the mediational analyses presented in the current study, including both childhood trauma, personality traits, and symptom distress, have not been reported in any previous publication.

### Measures

#### Childhood Trauma Questionnaire

This is a 25‐item self‐report inventory containing retrospective questions concerning childhood abuse and neglect (Bernstein *et al*., 2003). On a five‐point Likert‐type scale (‘Never true’ to ‘Very often true’) the respondent indicates how well each item describes how they feel about their childhood and teenage experiences. Research suggests that the Danish Childhood Trauma Questionnaire (CTQ) has acceptable psychometric properties (Kongerslev *et al*., 2019). In the current study, we computed a total composite score for childhood trauma by averaging all item responses, which is consistent with previous research (Bach & Fjeldsted, 2017). Cronbach's alpha value for the CTQ total scale was 0.94, and the mean corrected item‐total correlation was 0.61 (ranging from 0.39 to 0.79; median = 0.60).

#### Symptom Check List 27

Symptom Check List 27 (SCL‐27) is a 27‐item self‐report inventory capturing current internalizing symptomatology (within the last 7 days) including depressive symptoms, dysthymic symptoms, vegetative symptoms, agoraphobic symptoms, symptoms of social phobia, and symptoms of mistrust (Hardt & Gerbershagen, 2001). The SCL‐27 was originally extracted from the complete SCL‐90‐R (Derogatis, 1992), and was considered most suitable for the present study due to its exclusive emphasis on internalizing symptoms. In addition, it has been demonstrated that SCL‐27 is a sound measure of internalizing symptomatology such as depressive disorders (Prinz, Nutzinger, Schulz, Petermann, Braukhaus & Andreas, 2013). In the present study, we only used the total score (i.e., *General Severity Index [GSI]*) as an indicator of internalizing symptomatology. On the 27 five‐point Likert‐scale items (from [0] ‘not at all’ to [4] ‘extremely’) participants rated the intensity of symptoms within the past 7 days reflecting *state* features. In the present study, the Cronbach's alpha value for the GSI was 0.96, and the mean corrected item‐total correlation was 0.67 (ranging from 0.43 to 0.79; median = 0.68).

#### Personality Inventory for DSM‐5

This is a 220‐item self‐report inventory which we used to assess maladaptive personality traits (Krueger, Derringer, Markon, Watson & Skodol, 2012). The Personality Inventory for DSM‐5 (PID‐5) measures 25 trait facet scales organized into five higher‐order domain scales that correspond to the trait system of the DSM‐5 Alternative Model of Personality Disorders (AMPD): *Negative Affectivity*, *Detachment*, *Antagonism, Disinhibition*, and *Psychoticism*. The first four domains overlap with the ICD‐11 trait domain specifiers as well as universal Big Five traits (Bach *et al*., 2017; Gore & Widiger, 2013). The 220 four‐point Likert‐scale items were rated from ‘Very False or Often False’ to ‘Very True or Often True.’ There is an emerging base of empirical support for its structural validity, ability to capture personality pathology, and correspondence with various five factor models (Al‐Dajani, Gralnick & Bagby, 2016; Barchi‐Ferreira Bel & Osório, 2020; Pires, Sousa Ferreira & Guedes, 2017; Rogier, Beomonte Zobel & Velotti, 2020). Consistent with the definition of trait features and PDs, it has been supported that PID‐5 traits are highly stable over time and prospectively predict psychosocial functioning (Wright, Calabrese, Rudick *et al*., 2015). Research on the Danish PID‐5 supports its internal consistency, five‐factor structure, and criterion validity (Bach *et al*., 2017; Bach, Maples‐Keller, Bo & Simonsen, 2016; Bach & Sellbom, 2016; Bach, Sellbom, Bo & Simonsen, 2016; Bo, Bach, Mortensen & Simonsen, 2016) and its psychometric generalizability across clinical and nonclinical samples (Bach, Sellbom & Simonsen, 2018). In the present study, Cronbach's alpha values were 0.94 (Negative Affectivity; mean corrected item‐total correlation = 0.63); 0.95 (Detachment; mean corrected item‐total correlation = 0.65); 0.91 (Antagonism; mean corrected item‐total correlation = 0.55); 0.92 (Disinhibition; mean corrected item‐total correlation = 0.57); 0.96 (Psychoticism; mean corrected item‐total correlation = 0.62). Alphas for PID‐5 facet scales ranged from 0.73 (Irresponsibility) to 0.96 (Eccentricity).

### Statistical approach

Bivariate correlations among all primary study variables were computed followed by a series of multiple parallel meditation models based on Andrew Hayes' ‘Model 4’ (Hayes, 2013). First, the mediating effects of DSM‐5 trait domains were explored simultaneously. As a subsidiary analysis, the mediating effects of the most prominent PID‐5 facet scales were estimated simultaneously using a stepwise procedure: (1) meditational effects of facet scales were examined simultaneously for each domain; (2) the 10 most substantial mediators were designated and included in one combined parallel meditational model; and (3) facet scales with no significant effect sizes were sequentially excluded until the specific mediational effects of the remaining facet scales were significant.

In the mediation analyses we considered different effects. The total effect of an independent variable (IV) on a dependent variable (DV) is composed of the direct effect of the IV on the DV and the indirect effect through a proposed mediator variable. In the case of multiple parallel mediation, the total indirect effect of all proposed mediators and the specific indirect effect of each single mediator can be estimated. In the current study, we examined the direct and indirect effects of the CTQ score on the SCL‐27 score through the five PID‐5 domain scores, simultaneously. This procedure allows us to calculate the indirect effect of the individual mediator while controlling for the influence of other potential mediators. Based on recommendations by Hayes (2013) and MacKinnon, Fairchild and Fritz (2007), a bootstrapping sampling procedure (10,000 bootstrapped samples) was applied for assessing the indirect effects. Bootstrapping is a nonparametric approach that can provide more accurate inferences in particular when the data are not well behaved in terms of distributional assumptions (e.g., non‐normality) and when sample size is limited (Mooney & Duval, 1993). The reported coefficients and effects were considered significant if zero was not included in the 95% bias‐corrected confidence interval. All effects were adjusted for the influence of gender and age. In the current study we used PROCESS version 2.16.3 for SPSS provided by Hayes (2013).

## RESULTS

### Descriptive statistics and bivariate associations

Table 2 displays descriptive statistics and correlations among all study measures. Consistent with the first hypothesis, approximately all scales showed significant intercorrelations. Childhood Trauma was in particular related to the PID‐5 trait domains of Negative Affectivity and Detachment, which primarily were related to internalizing symptom distress. The correlations between Childhood Trauma and Antagonism as well as Antagonism and Symptom Distress were very weak, and the remaining associations with Antagonism were also rather weak.

**Table 2:** Intercorrelations, means, and standard deviations for all scales (*N* = 462)

Scale	1	2	3	4	5	6	Mean (*SD*)
1. CTQ: Childhood Traumas							1.65 (0.65)
2. SCL‐27: Internalizing Symptoms	0.56						0.92 (0.82)
3. PID‐5: Negative Affectivity	0.43	0.76					1.08 (0.67)
4. PID‐5: Detachment	0.53	0.70	0.57				0.79 (0.62)
5. PID‐5: Antagonism	0.12	0.13	0.16	0.22			0.65 (0.50)
6. PID‐5: Disinhibition	0.43	0.64	0.62	0.56	0.41		0.75 (0.54)
7. PID‐5: Psychoticism	0.44	0.66	0.58	0.56	0.46	0.67	0.57 (0.54)

*Note*: *N* = 462. All coefficients are significant at the 0.01 level, whereas coefficients from 0.16 are significant at the 0.001 level. CTQ = Childhood Trauma Questionnaire; PID‐5 = Personality Inventory for DSM‐5; SCL‐27 = Symptom Checklist 27.

### Mediational analysis

**Fig. 1:**
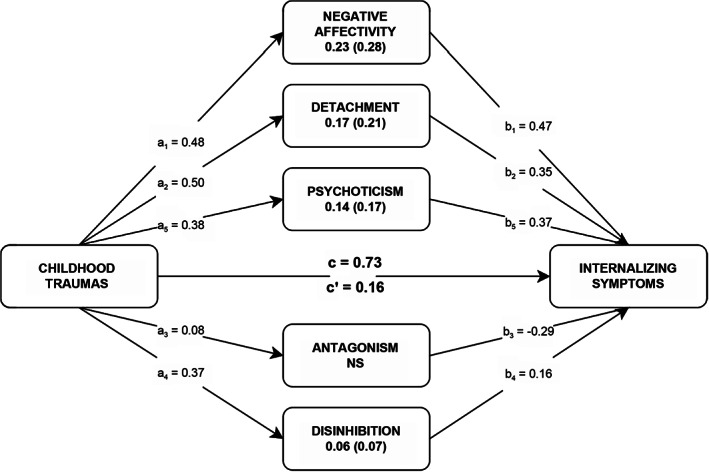
Paths linking Childhood Traumas to Internalizing Symptoms via PID‐5 Personality Trait Domains. *N* = 462. c = total effect, c′ = direct effect (when a and b paths are accounted for). NS = not significant. Estimations were statistically controlled for age and gender. All reported coefficients and indirect effects are significant in terms of 95% bias‐corrected confidence intervals (10,000 bootstrapped samples) that do not contain zero. Standardized mediational effects are reported in parentheses.

Childhood trauma explained 34% of the variance in internalizing symptom distress (total path coefficient of 0.73) when adjusting for the influence of age and gender. Results of multiple parallel mediation analysis for the PID‐5 traits are presented in Fig. 1. The direct effect (c′) was substantially reduced but remained significant. The total mediating effect of DSM‐5 traits (0.57) accounted for 78% of the total effect between childhood trauma and internalizing symptoms.

As shown in Fig. 1, only the PID‐5 trait domains of Negative Affectivity, Detachment, Psychoticism, and Disinhibition showed significant indirect effects while controlling for one another. The percentages of the total indirect effect were 40.4% (Negative Affectivity), 30.0% (Detachment), 24.6% (Psychoticism), and 10.5% (Disinhibition).

**Fig. 2:**
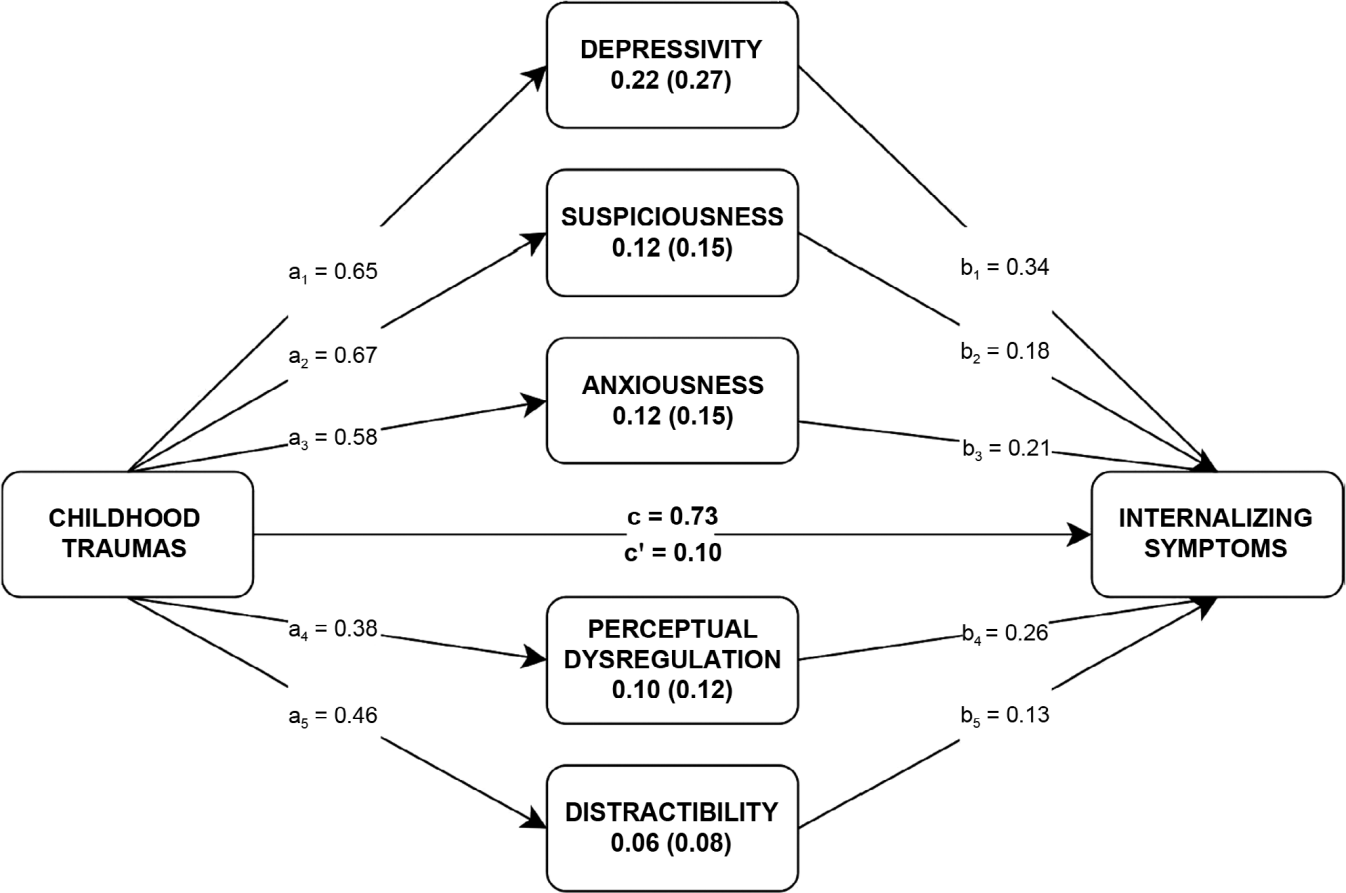
Lower‐Order PID‐5 Personality Traits that significantly mediate the effect of Childhood Trauma History on Internalizing Symptoms. *N* = 462. c = total effect, c′ = direct effect (when a and b paths are accounted for). Estimations were statistically controlled for age and gender. All reported coefficients and indirect effects are significant in terms of 95% bias‐corrected confidence intervals (10,000 bootstrapped samples) that do not contain zero. Standardized mediational effects are reported in parentheses.

As shown in Fig. 2, we further investigated the most essential lower‐order features of PID‐5 traits (i.e., facet scales) in terms of their meditational strengths. Accordingly, the most predominant PID‐5 trait facets were Depressivity, Suspiciousness, Anxiousness, Perceptual Dysregulation, and Distractibility.

## DISCUSSION

In the present study, we set out to test a proposed model in which PID‐5 traits mediate the effect of childhood traumas on internalizing symptom distress in adulthood. Overall, we found that childhood traumas explained 34% of the variance in symptom distress. The total indirect effect of PID‐5 trait domains accounted for 78% of the total effect of childhood traumas on internalizing symptoms. Although, these findings are based on statistical inferences from a cross‐sectional sample along with a single measurement method, it underscores the significant role of PID‐5 maladaptive personality traits (i.e., enduring liabilities) as potential mediators in the path between childhood adversities and current internalizing symptoms. Accordingly, the proposed mediational model may be a reasonable basis for more comprehensive investigation in future studies. Moreover, these preliminary results may support the value of systematically assessing psychological liabilities such as PID‐5 traits in traumatized patients with internalizing psychopathology.

Consistent with our primary hypothesis, the most substantial mediating effects applied to the PID‐5 trait domains of Detachment, Negative Affectivity, and Psychoticism. The PID‐5 trait domain of Disinhibition showed a significant but very modest indirect effect, whereas the effect of Antagonism was non‐significant. The substantial indirect effects of Negative Affectivity, Detachment, and Psychoticism suggest that features of these domains may be particularly related to both traumas and internalizing disorders.

As a subsidiary analysis, we also identified the most predominant lower‐order features of PID‐5 traits in terms of meditational effect. This involved the PID‐5 trait facets of Depressivity, Anxiousness, Perceptual Dysregulation, Suspiciousness, and Distractibility. These findings are largely consistent with previous research on mediators between childhood adversity and adult psychopathology. For example, previous studies suggest that mistrust (Jenkins, Meyer & Blissett, 2013), depressive traits (Whiffen, Parker, Wilhelm, Mitchell & Malhi, 2003), and anxiousness (Wanner, Vitaro, Tremblay & Turecki, 2012) comprise substantial mediators in this relationship. Most important, the findings are particularly consistent with the study by Veith *et al*. (2017) who also found Negative affectivity and Detachment to mediate the effect of childhood trauma on internalizing symptoms. Interestingly, the substantial mediating role of Psychoticism, including the facet of Perceptual Dysregulation (i.e., dissociation proneness), is consistent with research showing that dissociation (i.e., depersonalization and derealization) mediates between child sexual abuse and later non‐suicidal self‐harm (Bach & Fjeldsted, 2017; Swannell, Martin, Page *et al*., 2012). In the present study, we were not able to explain the mediating role of Distractibility, however, the effect of this facet may possibly be accounted for by enduring neuro‐cognitive deficits, poor concentration, and chaotic thinking related to symptom distress, or trauma‐related distractions including flashbacks.

### Consideration of clinical implications

As would have been expected, the PID‐5 traits demonstrated a potential ability to mediate the effect of childhood traumas on internalizing symptomatology consistent with previous meditational research on the DSM‐IV PD model now retained in DSM‐5 Section II (Johnson, Quigley & Sherman, 1997; Kounou *et al*., 2013; Waller, 1993). Accordingly, clinicians should not only pay attention to symptoms of psychopathology but also the underlying personality‐related potential for psychopathology. As stated by Osler (1899), ‘It is more important to know the person with the illness than the illness the person has.’ For example, in psychotherapy for patients with childhood traumas and current internalizing symptoms, this could involve targeting personality dysfunction associated with Negative Affectivity (e.g., anxiousness and suspiciousness) and Detachment (e.g., social withdrawal and lack of positive affectivity). This could be implemented by means of transdiagnostic treatment focusing on such traits as common features driving various emotional disorders including PDs (Bach & Presnall‐Shvorin, 2020; Sauer‐Zavala, Bentley & Wilner, 2016). This is particularly relevant for the novel ICD‐11 Personality Disorders and Related Traits where Negative Affectivity and Detachment comprise trait domain specifiers that may be coded by the clinician (Bach & First, 2018). Finally, the considerable meditational effect of PID‐5 Psychoticism may suggest that possible features of perceptual dysregulation or dissociation should be prioritized in the treatment of anxious and depressed individuals suffering from childhood traumas, which is consistent with psychotherapy research showing that the presence of dissociation predicts avoidance, treatment discontinuation, and lack of recovery (Arntz, Stupar‐Rutenfrans, Bloo, van Dyck & Spinhoven, 2015).

### Limitations and future directions

The findings of this study should be interpreted in light of certain limitations, which point at directions for future research. First, the employment of mediation analysis on cross‐sectional data has been questioned because there can be found evidence for indirect effects even when the true indirect effect in longitudinal data would be zero, and vice versa (Maxwell & Cole, 2007). Nevertheless, in the present study mediation analysis was used in accordance with recommendations by Hayes (2013) and MacKinnon *et al*. (2007) as a preliminary attempt to test a proposed model. Moreover, our goal was also to compare the mediational effects exerted by five different PID‐5 personality trait domains and their subfacets. Thus, the findings provided initial support for the proposed model, but longitudinal research is warranted for a conclusive test of mediation.

Second, because childhood traumas were self‐reported, we were not able to differentiate between perceived and actual childhood events. It is plausible that the findings would have been different if reports from multiple informants (e.g., parents, siblings) had been accessible. Moreover, the retrospectively recollected reporting may have been influenced by recall bias and state‐dependent memory reflecting both personality and current mental condition. However, research suggests that retrospective self‐reports and prospective records of childhood maltreatment are highly comparable, which support the utility of self‐reported childhood adversities (Scott, McLaughlin, Smith & Ellis, 2012).

Third, both PID‐5 traits and internalizing symptoms were almost concurrently self‐reported involving a risk for artificially high correlations among measures (Campbell & Fiske, 1959). More definitive results would likely have been achieved if it had been possible to administer structured clinical interviews for all the participants at different points in time. For the DSM‐5 and ICD‐11 traits, this could also include clinician or informant ratings (Bach, Christensen, Kongerslev, Sellbom & Simonsen, 2020; Markon, Quilty, Bagby & Krueger, 2013).

Finally, we recommend future studies in developmental psychopathology to focus on the DSM‐5 and ICD‐11 component of global personality dysfunction (Bach, Brown, Mulder, Newton‐Howes, Simonsen & Sellbom, 2021; Zimmermann, Müller, Bach, Hutsebaut, Hummelen & Fischer, 2020), which appears to be more malleable than stylistic trait expressions (Bach & Simonsen, 2021; Back *et al*., 2021; Gander, Buchheim, Bock, Steppan, Sevecke & Goth, 2020; Wright, Hopwood, Skodol & Morey, 2016).

## Data Availability

The data that support the findings of this study are available from the corresponding author upon reasonable request.
